# *Quillaja lancifolia* Immunoadjuvant Saponins Show Toxicity to Herbivores and Pathogenic Fungi

**DOI:** 10.3390/plants14081252

**Published:** 2025-04-20

**Authors:** Anna C. A. Yendo, Luana C. Colling, Hélio N. Matsuura, Lúcia R. B. Vargas, José A. Martinelli, Gabriela Z. Chitolina, Marilene H. Vainstein, Arthur G. Fett-Neto

**Affiliations:** 1 Plant Physiology Laboratory, Center for Biotechnology and Department of Botany, Federal University of Rio Grande do Sul (UFRGS), Porto Alegre 91501-970, RS, Brazil; anna.yendo@gmail.com (A.C.A.Y.);; 2 Solubio-Rodovia GO 184 Km 09 à Direita, Trevo com a Rodovia JTI 101-S/N Zona Rural-Jataí/GO, Jataí 75800-000, GO, Brazil; 3 Department of Plant Pathology, Federal University of Rio Grande do Sul (UFRGS), Porto Alegre 91540-000, RS, Brazil; 4 Laboratory of Fungi of Medical and Biotechnological Importance, Center for Biotechnology, Federal University of Rio Grande do Sul (UFRGS), Porto Alegre 91501-970, RS, Brazil

**Keywords:** antifungal, antiherbivore, saponin, specialized metabolism, *Quillaja*

## Abstract

Saponins from leaves of *Quillaja lancifolia*, a native species from southern Brazil, show potent immunoadjuvant activity in experimental vaccine formulations. The accumulation of the immunoadjuvant saponin fraction QB-90 is induced in cultured leaf disks and seedlings by several stresses and stress signaling molecules, such as osmotic agents, salicylic acid, jasmonic acid, mechanical damage, ultrasound, UV-C radiation, and high light irradiance. These observations suggest a role in plant defense. To further examine this possibility, an investigation of the potential inhibitory role of *Q. lancifolia* saponins on plant and human pathogenic fungi and two herbivore models was carried out. The screening tests showed that saponin-rich fractions, particularly QB-90, were able to significantly inhibit the growth of *Bipolaris micropus*, *Curvularia inaequalis*, *Fusarium incarnatum*, and *Cryptococcus gattii* R265. The same metabolites acted as deterrents against the generalist mollusk and insect herbivores *Helix aspersa* and *Spodoptera frugiperda*, respectively. Significant reductions in consumption of leaf area and larvae body weight were recorded. Taken together, these data indicate a role for *Q. lancifolia* saponins in plant defense against diverse herbivores and fungi, having potential as a natural pest control agent and/or as a molecular platform for the development of new environmentally friendly biocide molecules.

## 1. Introduction

Insect pests and plant pathogens are a major source of losses in the production of agricultural crops. Combined, these agents account for approximately 38% of global agricultural losses, mainly in developing countries [1]. Preventive and remedial herbivore control measures must be incorporated in crop production systems to reduce losses. Considering pathogens, fungal diseases are a major limiting factor in crop production [2]. These diseases have been controlled by synthetic chemical fungicides in modern agriculture. Excessive and frequent use of pesticides has led to the development of resistance among pathogens and herbivores, as well as environmental contamination, which is of potential risk to animal and human health. In recent decades, possibly due to climate change, insects have been shifting their distribution ranges in complex patterns [3], often inflicting more harm to crops previously unexposed to such foes. These problems highlight the need to develop alternative methods for controlling plant pests.

A variety of plant specialized metabolites can act as defenses against herbivores and pathogens, including alkaloids, flavonoids, terpenes, glucosinolates, and phenolic acids [4]. Saponins are glycosylated terpenes which often show defense properties. Saponins are widely distributed in plants, characterized by a complex structure based on a skeleton derived from a 30-carbon 2,3-oxidosqualene precursor that has one or more sugar residues [5,6]. As plant specialized metabolites, their accumulation is often stimulated in response to environmental challenges mediated by biotic or abiotic stresses, such as high and low temperatures, drought, salinity, ultraviolet radiation or ozone stress, herbivory, and pathogen infections. These responses are mediated by specific receptors and signaling molecules, such as jasmonic acid, methyl jasmonate, salicylic acid, methyl salicylate, and nitric oxide, which have been shown to take part in defense signaling against herbivore and pathogen attacks [7]. The number of studies showing the activity of concentrated saponin fractions or pure saponins against insects is considerable, including aphids, beetles, caterpillars, and flies [8]. Additionally, enhancement of saponin concentration upon jasmonate exposure can be observed in several plant species, including *Glycyrrhiza glabra* and *Centella asiatica* [9,10], supporting the role of these metabolites in defense against herbivores.

*Quillaja lancifolia* D.Don (Quillajaceae), formerly *Quillaja brasiliensis* (A.St.-Hil. & Tul.) Mart., is a saponin-producing tree native to southern Brazil [11]. This plant is commonly known as soap tree, due to the capacity of its leaves and barks to produce persistent foam in water. Purified saponin fractions from its leaves, namely QB-90 and QB-80, are able to stimulate humoral and cellular immune responses in mice against different virus types, such as *herpesvirus* type 1 and 5, poliovirus, rabies virus, and bovine viral diarrhea virus, in a comparable manner to the commercial saponin fraction Quil-A, which is extracted from the barks of a related species, *Q. saponaria*, native to Chile [12]. Previous studies examining the effects of abiotic or biotic stresses on the accumulation patterns of QB-90 proved that its amount was significantly increased by different osmotic stress agents, upon exposure to salicylic acid, jasmonic acid, leaf mechanical damage, ultrasound, increased white light irradiance, and UV-C [13]. QBs and some related fractions have been analyzed by mass spectrometry. These evaluations revealed several triterpene saponins with aglycones of the β-amyrin group, mainly quillaic acid [14,15]. Some of the common saponin structures found in *Q. lancifolia* are shown in Figure S1. Among the saponins found in QB fractions are the immunogenic QS-21 and QS-7 [16].

Considering that increased accumulation of these terpenes may be part of a defense response, and that both insect pests and fungal diseases are major causes of losses in agriculture, the effect of *Q. lancifolia* saponins as potential inhibitors of two herbivore models *Spodoptera frugiperda* (Smith) (Lepidoptera: Noctuidae) and *Helix aspersa* (Stylommatophora: Helicidae) was investigated. In addition, possible antifungal activity was evaluated against phytopathogenic (*Bipolaris micropus*, *Curvularia inaequalis* and *Fusarium incarnatum*) and human pathogenic (*Cryptococcus neoformans grubi H99*, *C. neoformans neoformans B3501 and C. gattii R265*) fungi. This study addressed whether *Q. lancifolia* saponins represent a possible alternative and environmentally friendly strategy for herbivore and pathogenic fungal control. Potential use against human pathogenic fungi was also examined. Finally, this investigation also sought a better understanding of the potential functions of these saponins in planta.

## 2. Results

### 2.1. Antifungal Assays

The saponin-based treatments were evaluated for their antifungal properties against three phytopathogenic species and two human pathogenic species, all of them of significant agricultural and health importance. Antifungal assays for phytopathogens were performed by measuring fungal growth diameter under different treatments. After 4 days of experiments, none of the aqueous extract treatments and saponin fractions showed a significant difference in inhibition of the analyzed fungi. The positive control was effective in inhibiting fungal growth, whereas normal growth was observed in the negative control. After 7 days of experiments, however, significant inhibition of fungal growth was observed in the presence of saponins, at least in some of the concentrations and fractions tested. In addition, some differences in the pattern of inhibition between the analyzed microorganisms were also recorded.

**Figure 1:**
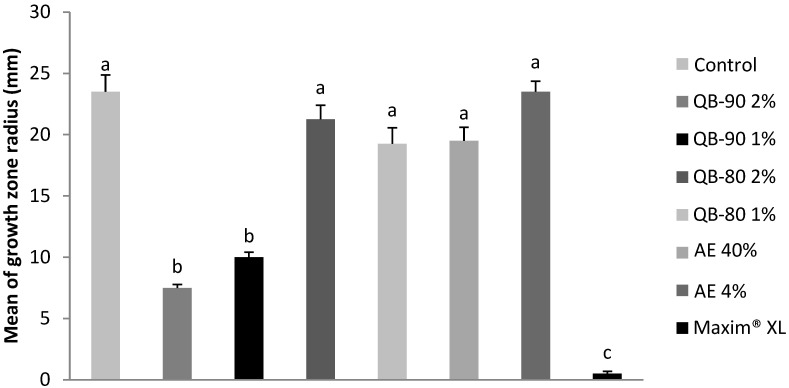
Growth radius of the fungus *B. micropus* after 7 days of incubation in agar containing AE or saponin fractions from *Q. lancifolia.* Treatments were formulated with fractions (*m*/*v*) QB-90 2% or 1%, QB-80 2% or 1%, AE 40% or 4%, Maxim^®^ XL 1 ppm (positive control), or water (negative control). Bars represent mean ± standard error. Different letters indicate significant difference by Tukey test (*p* ≤ 0.05).

*Bipolaris micropus* showed growth inhibition when subjected to treatment with one of the saponin fractions, QB-90. QB-90 at 1% and 2% inhibited fungal growth by 58% and 68%, respectively (Figure 1). In contrast, QB-80 fraction and aqueous extract were not able to significantly inhibit fungal growth.

**Figure 2:**
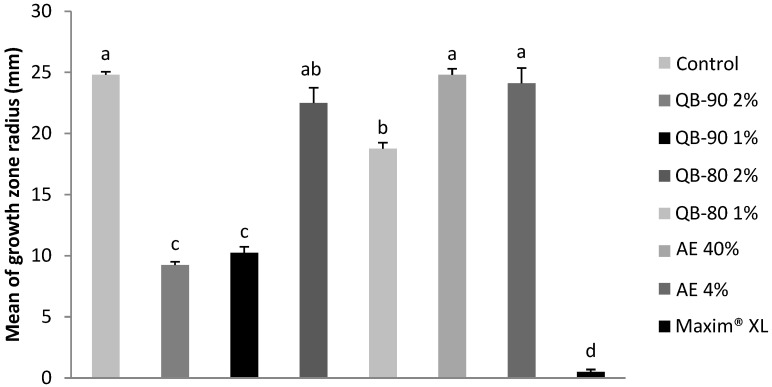
Growth radius of the fungus *C. inaequais* after 7 days of incubation in agar containing AE or saponin fractions from *Q. lancifolia.* Treatments were formulated with fractions (*m*/*v*) QB-90 2% or 1%, QB-80 2% or 1%, AE 40% or 4%, Maxim^®^ XL 1 ppm (positive control), or water (negative control). Bars represent mean ± standard error. Different letters indicate significant difference by Tukey test (*p* ≤ 0.05).

In a similar fashion, growth of *Curvularia inaequalis* was inhibited by 59% and 63% by the treatments QB-90 1% and 2%, respectively. Treatment with QB-80 had a minor inhibitory effect compared to QB-90. Treatments with aqueous extracts did not inhibit growth (Figure 2).

**Figure 3:**
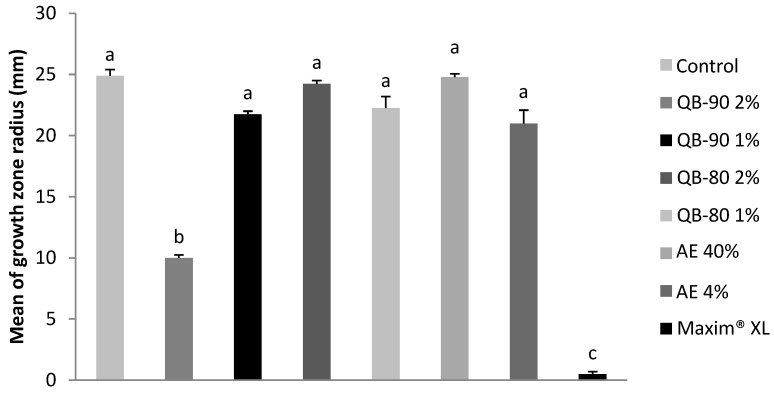
Growth radius of the fungus *F. incarnatum* after 7 days of incubation in agar containing AE or saponin fractions from *Q. lancifolia.* Treatments were formulated with fractions (m/v) QB-90 2% or 1%, QB-80 2% or 1%, AE 40% or 4%, Maxim^®^ XL 1 ppm (positive control), or water (negative control). Bars represent mean ± standard error. Different letters indicate significant difference by Tukey test (*p* ≤ 0.05).

Differently from the other phytopathogenic fungi, a dose-dependent effect on growth inhibition of the fungus *Fusarium incarnatum* was recorded. The highest concentration of QB-90 inhibited growth of *F. incarnatum* by 60%, whereas the lowest concentration of the same fraction of saponin had no significant effect (Figure 3). QB-80 fraction and extracts were also not significantly inhibitory (Figure 3).

**Figure 4:**
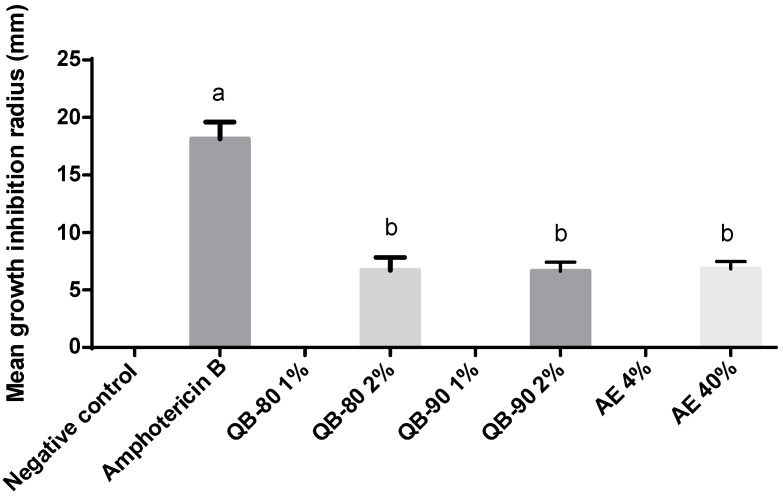
Fungi growth radius inhibition for *C. gattii* R265 after 24 h of incubation with treatment-impregnated disks containing EA or saponin fractions from *Q. lancifolia.* Treatments were formulated with fractions (*m*/*v*) QB-90 1% or 2%, QB-80 1% or 2%, AE 4% or 40%, amphotericin B 0.2% (positive control), or water (negative control). Absence of bars indicates no inhibition. Bars represent mean ± standard error. Different letters indicate significant difference by Tukey test (*p* ≤ 0.05).

**Figure 5:**
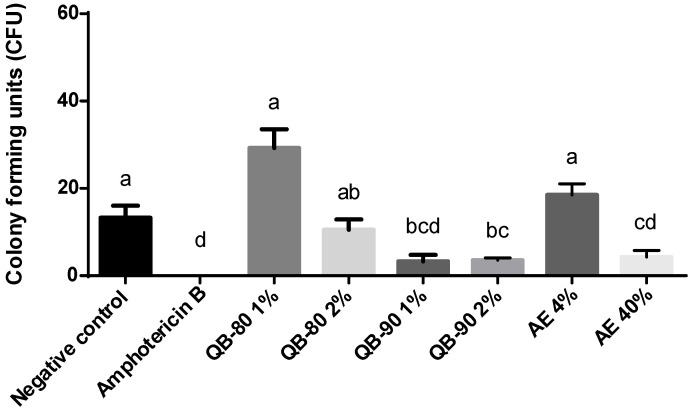
Number of colony forming units (CFUs). After 24 h incubation of *C. gattii* R265 in liquid medium containing EA or saponin fractions from *Q. lancifolia*, samples were diluted to 1 × 10^4^ cells. An aliquot of 100 µL of each of the suspensions was separately applied to solid growth medium and counting was carried out after 48 h. Treatments were formulated with fractions (*m*/*v*) QB-90 1% or 2%, QB-80 1% or 2%, AE 4% or 40%, amphotericin B 0.2% (positive control), or water (negative control). Absence of bars indicates full inhibition. Bars represent mean ± standard error. Different letters indicate significant difference by Tukey test (*p* ≤ 0.05).

*Q. lancifolia* saponin fractions QB-80, QB-90 and the saponin-rich aqueous extract had no antifungal activity against *C. neoformans grubi* and *C. neoformans neoformans*. On the other hand, the saponin-based treatments significantly inhibited *C. gattii* growth. In fact, both QB-80 and QB-90 at 2%, and AE at 40%, inhibited fungal growth by approximately 40% in comparison to the positive control (Figure 4). After 24 h incubation of *C. gattii* R265 in medium containing treatment solutions and 48 h incubation of the diluted fungal aliquot, the colony forming units (CFU) count showed that AE 40% inhibited fungal growth by 68%. In contrast to the radius growth inhibition test, QB-80 2% showed only a trend of decreasing the number of CFUs (by circa 20%) (Figure 4 and Figure 5). On the other hand, QB90 1% had no effect in the radius growth assay but was able to inhibit CFUs in the corresponding test. Indeed, QB-90 1% and 2% were equally effective in inhibiting fungal growth as CFUs (74%), without a dose-dependent profile (Figure 5).

### 2.2. Generalist Herbivore Assays

**Figure 6:**
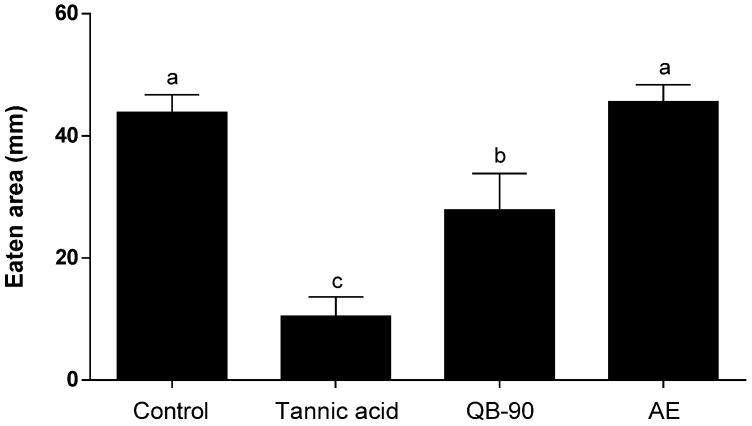
Leaf area consumed by *H. aspersa*. Lettuce disks were treated with methanol (negative control), tannic acid 88.17 mM (positive control), QB-90 fraction 2 mg.mL^−1^, or AE 40 mg.mL^−1^ from *Q. lancifolia*. Bars represent the means ± standard error. Different letters indicate significant difference by Tukey test (*p* ≤ 0.05).

Deterrent assays with generalist herbivores were performed, offering to snail *H. aspersa* and caterpillar *S. frugiperda* diets impregnated with treatments containing saponins. In both cases, the addition of saponin-enriched fraction affected the feeding behavior of the animals. Snails exposed to QB-90 diet consumed less lettuce disks than the negative control group. QB-90 was able to significantly inhibit feeding by 36% compared to the negative control, although the saponin fraction deterrence capacity did not surpass that of tannic acid (positive control). On the other hand, AE failed to inhibit feeding (Figure 6). No snails died during the experimental period.

**Figure 7:**
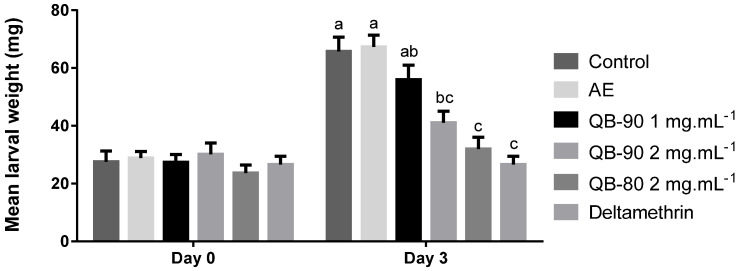
Weight of *S. frugiperda* larvae feeding for three days on a diet containing AE or saponin fractions of *Q. lancifolia*. Diet preparation was formulated with saponin fractions QB-80 2 mg.mL^−1^ or QB-90 1 or 2 mg.mL^−1^, AE 40 mg.mL^−1^, Deltamethrin 0.1 mg.mL^−1^ (positive control), or methanol 30% (negative control). Larval weights did not differ significantly at the start of the experiment. Bars represent the means ± standard error. Different letters indicate significant difference by Tukey test (*p* ≤ 0.05).

Larvae of *S. frugiperda* fed with meal treated with saponin-enriched fractions had reduced weight gain after three days of experiment (Figure 7). On diets with high concentration of saponin fractions (QB-80 and QB-90 at 2 mg.mL^−1^), the larval weight was reduced by 51% and 38%, respectively. AE did not inhibit caterpillar feeding and weight gain. Interestingly, the QB-90 response profile was observed throughout the experiment, starting at day 1 (Figure S2). Significant larvae deaths were only recorded in the positive control treatment (Deltamethrin).

## 3. Discussion

Saponins from leaves of *Q. lancifolia* inhibited the growth of three phytopathogenic fungi and had selective inhibitory activity on *C. gattii*, while not presenting effects against *C. neoformans grubi* and *C. neoformans neoformans*. The observed responses confirmed the antifungal action of the saponins present in the enriched fraction, as expected for this class of metabolites [17]. Overall data are consistent with previous studies that pointed to saponins as metabolites with relatively broad antifungal activity.

As pointed out above, there were occasional discrepancies in the inhibitory activity detection of saponin fractions (e.g., QB-90 1% and QB-80 2%) between the CFU counting and the halo inhibition methods used in the evaluation of *C. gattii*. This could have been due to the differences in exposure time between the methods and/or diffusion of components of the saponin fractions. Exposure to AE failed to inhibit phytopathogenic fungi and herbivore feeding, possibly due to the low specific concentration of deterrent saponins present in the extract. However, growth of *C. gattii* was inhibited by AE 40% in both types of assays (radial development and colony formation). This result should be viewed with caution, since osmotic effects of the high AE concentration may have contributed to the negative impact on this target species.

Extracts of saponin-rich plants such as *Balanites aegyptiaca*, *Quillaja saponaria*, and *Yucca schidigera* have been shown to inhibit the growth of plant pathogenic fungi [18]. Triterpenoid saponins from *Chenopodium quinoa* protected tomato plants against *Fusarium* wilt infection [19]. The triterpenic saponin aescin displayed strong inhibitory effect on mycelial growth of fungal isolates from *Leptosphaeria maculans*, *Microdochium nivale*, and *Pyrenophora teres* [20]. Aescin exhibited EC_50_ values below 50 µg.mL^−1^ against these pathogens. Other extracts rich in these secondary metabolites, including triterpenic and steroidal saponins, showed fungicidal effect against several human pathogenic fungi species, such as *C. neoformans*, *Candida albicans*, *Aspergillus fumigatus*, *Pneumocystis carinii*, *Trichophyton rubrum*, *Epidermophyton floccosum*, and *Microsporum gypseum* [21,22,23,24,25,26,27]. Several phytopathogenic fungi, including *Venturia inaequalis*, *Botrytis cinerea*, *Magnaporthe oryzae*, *Plasmopara viticola*, *Fusarium kuroshium*, *Penicillium digitatum*, *Rhizopus stolonifera*, *Valsa mali*, *Botryosphaeria dothidea*, and *Alternaria alternata*, were also inhibited by saponin-rich extracts [28,29,30,31,32,33].

A particular extract rich in saponins can have varied antifungal activity against different fungi. For example, extract of *Balanites aegyptiaca* had high inhibitory activity against *Pythium ultimum* and mild stimulant activity against *Colletotrichum coccodes*. This variation may be related to the sterol and lipid composition of the cell membranes of different fungi [18]. In fact, this feature was pointed as a possible cause of the different sensitivities of phytopathogenic fungi (e.g., *Alternaria solani*, *Botrytis cinerea*, *Fusarium sambucinum*, *Pythium sulcatum*) to the antimicrobial cyclic lipopeptide fengicin from *Bacillus subtilis* CU12; in this case, a relevant role was attributed to differences in the concentration of ergosterol and anionic phospholipids [34]. *Q. saponaria* bark saponins are known to interact with membrane components [35]. It is expected that the same occurs with *Q. lancifolia* leaf saponins, which are structurally very similar to those of the bark of the Chilean species [12].

The association of saponins, mainly with sterols, can result in complexes that alter the integrity of the fungal cell membrane and/or cause the formation of transmembrane pores. The fungicidal activity of saponins may also be related to their structure, as the number, type, and sequence of their sugar residues have a great effect on bioactivity [21,36]. In fact, in oleanolic acid-type saponins isolated from *Pulsatilla chinensis*, a C-28 free carboxyl, a C-23 hydroxyl group, and a C-3 oligosaccharide chain proved essential for antifungal activity [27].

Considering the growing increase in plant diseases caused by phytopathogenic fungi, coupled with environmental and resistance problems generated by the misuse of chemical fungicides, the development of new tools to control these pathogens becomes necessary. A good alternative to conventional fungicides is the use of those of natural origin, since they may have less toxicity to other organisms and a low residual effect in the environment due to biodegradability. As observed in this study, however, natural fungicide’s spectrum of action is not always homogeneous for different fungi, indicating the need for specific uses. Nonetheless, the three phytopathogens examined in this work were significantly inhibited by the highest concentration of QB-90 evaluated (2% *w*/*v*). A dose-dependent effect was observed in previous studies on the bioactivity of saponins derived from other plant species [18,21].

To sum up, 2% and 1% QB-90 fractions showed the best growth inhibition results against *B. micropus* and *C. inaequalis*. The highest concentration of the same fraction was also significantly inhibitory for *F. incarnatum*. However, the 1% QB-80 fraction showed activity only against *C. inaequalis*. The level of growth inhibition was often dependent on the type of saponin fraction analyzed. Although the two QB saponin fractions share several compounds, they are not identical. However, it is unclear at this point which differences in composition may have caused the specific effects on the tested pathogens. It is possible that small differences in the saponins present in each fraction may have interfered with their specific bioactivity [18]. In contrast, the toxic effect of QB-90 and QB-80 on larvae of *S. frugiperda* was comparable, possibly due to the major differences expected in cell membrane composition between fungi and arthropods.

At least for the time course used in the present experiments, saponin-induced inhibition of phytopathogenic fungal development was more effective at late stages (7 days) compared to early ones (4 days). This may be due to the putative mechanism of action of saponins by disruption of membrane integrity and function, which may be intensified by some degree of active growth and new membrane assembly. Taken together, the results indicate a defense role of saponins from *Q. lancifolia* against fungi and their potential as chemical agents in the control of phytopathogenic fungi.

Considering the growing problem of insect resistance to several groups of insecticides, the development of new pesticides is an emergent issue. Plant-derived toxins against insects can be a good alternative to synthetic chemical products, since they frequently display lower mammal toxicity and/or reduced residence period in the environment. Plant-derived feeding deterrents may also be advantageous due to their effects on herbivore feeding behavior and relatively narrower activity spectrum, thereby increasing the chances of preserving beneficial insects or the natural enemies of herbivores. From an analogous viewpoint, one might consider the interesting possibility that the lack of antifungal activity of some of these saponins (e.g., QB-80) may be an asset for the maintenance of beneficial fungi, such as mycorrhizae and endophytes, in the process of controlling herbivores.

A protective effect against insects has been recorded for saponins present in several plant families and species, such as *Medicago sativa*, *M. truncatula*, *Ricinus communis*, *Glycine max*, *Q. saponaria*, *Glycyrrhiza glabra*, and *C. quinoa* [37,38,39,40]. Saponins have shown insecticidal activity against caterpillars, beetles, mosquitoes [41,42,43], and aphids [8]. Survival decreases and effective deterrence of the aphid *Acyrthosiphon pisum* caused by saponins have been recorded [44]. Protodioscin saponin from *Gypsophila*, as well as *Camellia sinensis* and *Q. saponaria* saponins, showed significant toxicity towards *Helicoverpa zea*, *H. armigera*, and *S. frugiperda* [38,45].

The concentration of saponins can be modulated by herbivory injury. Indeed, QB-90 accumulation increased in *Q. lancifolia* leaves after treatment with the herbivory signaling hormone jasmonic acid or mechanical damage simulation [13]. In a similar fashion, damage by *Spodoptera littoralis* caterpillars increased alfafa foliar saponin levels [37]. These results further support the role of saponins as protection metabolites against herbivores, in good agreement with their possible roles as deterrents, toxins, and digestibility inhibitors.

From a practical viewpoint of pest control, *Q. lancifolia* saponins have some advantageous features. The species can be easily propagated from cuttings and cultivated in extensive miniclonal gardens, yielding large amounts of leaf biomass year-round in sustainable fashion [12]. Some of the obvious advantages of these metabolites include water solubility, biodegradability, and low toxicity to humans and other mammals, as shown by the use as vaccine adjuvants. Besides the antifungal and anti-insect properties herein described, the same saponins of *Q. lancifolia* have been proven effective as potential bioherbicides [14], featuring multiple pesticide activities. Based on immunoadjuvant bioactivity data, once dried by low heat or lyophilization, *Q. lancifolia* saponins remain stable for several months and can be stored at room temperature under dry environment. Similarly, QB-90 concentrations in harvested leaves left to dry for 10 days at room temperature remain stable [46]. The surfactant properties of triterpene saponins afford easy adaptation to application with ordinary spraying devices. Finally, the capacity to readily form nanocapsules make *Q. lancifolia* saponins ideal for combined application with other bioactive products [47] and/or biostimulants, providing a means of stabilization, increased activity at lower concentration, and slow release (i.e., agro-nanotechnology) [48].

## 4. Materials and Methods

### 4.1. Plant Material and Preparation of Aqueous Extract and Saponin-Enriched Fractions QB-80 and QB-90

*Q. lancifolia* leaves were collected from adult plants growing in the city of Canguçu, RS, Brazil (31°23′42″ S-52°40′32″ W). A voucher specimen was deposited at the ICN Herbarium of the Federal University of Rio Grande do Sul (142953). Harvest authorization was granted by the Brazilian Board of Management of Genetic Resources—CGEN (A98B7B1). Leaves were air-dried in plastic trays kept at room temperature (25 ± 3°C) under shade for 2 weeks. Air-dried leaves were ground to a fine powder with a mortar and pestle. Next, leaf powder was extracted in distilled water (1:10, *w*/*v*) for 8 h with magnetic stirrer agitation, and then vacuum-filtered through qualitative filter paper. Tannins were precipitated with 2% (*w*/*v*) gelatin; after additional filtration, aqueous extract was partitioned with reagent grade ethyl acetate (1:1 *v*/*v*). After discarding the organic fraction, the aqueous fraction was fully dried in a rotary evaporator at a temperature below 45 °C, yielding the aqueous extract (AE). Approximately 1 g of AE was resuspended in the smallest possible volume of water and submitted to purification through C18 reverse-phase silica gel chromatography and gradient of water and methanol to obtain fraction QB-90 (eluting at 90% methanol), as previously described [49]. QB-80 (eluting at 80% methanol) was obtained using the same protocol. QB-80 and QB-90 were also analyzed by TLC to further confirm fraction isolation.

### 4.2. Antifungal Assays

#### 4.2.1. Fungi Cultures

Phytopathogenic fungi *Bipolaris micropus*, *Curvularia inaequalis* and *Fusarium incarnatum* were provided by the Laboratory of Winter Cereals, Department of Plant Pathology, UFRGS. Fungal cultures were maintained in PDA medium (potato 140 g.L^−1^, dextrose 10 g.L^−1^, agar 20 g.L^−1^) with controlled temperature (25 ± 3 °C), under a 12 h light/dark cycle.

Human pathogenic fungi *Cryptococcus neoformans* grubi strain H99, *C. neoformans* neoformans strain B3501 and *C. gattii* strain R265 were grown in the Laboratory of Fungi of Medical and Biotechnological Importance, Center for Biotechnology, UFRGS. Human pathogenic fungal cultures were maintained in MH agar medium (g/L distilled water: 2 beef extract, 17.5 casein acid hydrolysate, 1.5 starch, 17 agar) at 35 °C, in the dark.

#### 4.2.2. Fungal Bioassays

For phytopathogenic fungi assays, the agar diffusion method was used [50]. Briefly, 200 µL of saponin-based AE (4% and 40% (*w*/*v*)), QB-80 (1% and 2%, *w*/*v*) and QB-90 (1% and 2%, *w*/*v*), negative control (distilled water), and positive control (Maxim XL^®^—1 ppm) were added to separate Petri dishes (25 mm radius) containing solidified PDA medium and allowed to dry. A 2 mm diameter plug of the actively growing mycelium of each fungi was placed in the center of each plate. The plates were inverted and incubated at 25 ± 3 °C under a 12 h light/dark cycle (4 plates per treatment, per fungus), and the growth diameters were measured in millimeters after 4 and 7 days of incubation. Assays were repeated twice independently.

For human pathogenic fungi, two methodologies were used: measurement of the halo inhibition of impregnated disks with the test solutions (disc diffusion technique) [50] and counting of the colonies grown after exposure of the fungus to the medium containing treatments (CFU assay) [51]. For the first methodology, Saboraud agar plates were inoculated with 1 × 10^7^ cells of the yeast using a Drigalski handle. The assay was performed by dispensing filter paper impregnated with saponin-based AE (4% and 40% *w*/*v*), QB-80 and QB-90 (1% and 2% *w*/*v*), distilled water (negative control), or Amphotericin B 0.2% (positive control) solutions onto the surface of the inoculated agar plates (4 replicates per treatment, per yeast). The plates were inverted and incubated at 30 °C (dark), and growth inhibition diameters were measured after 24 h. For the counting of the colony grown methodology, 50 μL of medium containing 1 × 10^7^ cells of the yeast were added to 96-well plates with 50 μL of each of the same treatments described for the halo inhibition methodology and incubated at 30 °C for 24 h. Afterwards, dilutions to 1 × 10^4^ cells of these inoculum suspensions were prepared and 100 µL of the suspension were transferred to Saboraud solid growth medium for 48 h, after which number of CFUs was determined. Replicate was performed by counting in 4 different quadrants.

### 4.3. Herbivore Assays

#### 4.3.1. *Helix aspersa* Assay

*H. aspersa* snails (Stylommatophora: Helicidae) were collected in Porto Alegre, RS, Brazil, and maintained until assay on a transparent plastic container (30 × 15 × 12 cm) divided into two chambers. For the experiment, each chamber received a small water container and one individual of *H. aspersa* weighing between 6 and 10 g (approximately between 3 and 6 months of age). The snails were starved for 24 h at 18 °C under a photoperiod of 16 h.day^−1^ provided by white fluorescent lamps prior to the experiment. The tests were based on a previously published protocol [52], with minor modifications. Assays were performed using the no-choice method, with 10 replicates per treatment and two independent experiments. Sixteen lettuce disks (2 cm diameter) per sample were treated with 32.89 μL of 2 mg.mL^−1^ QB-90 fraction, 40 mg.mL^−1^ *Q. lancifolia* leaf aqueous extract, 8.82 mM tannic acid (positive control), or methanol (negative control). Upon total evaporation, corresponding treatment disks were offered to snails. Snails were kept for 48 h at 18 °C, 16 h light photoperiod. Leaf-disk eaten area was measured with millimeter graph paper and processed by ImageJ software (version 1.53c).

#### 4.3.2. *Spodoptera frugiperda* Assay

*S. frugiperda* (Lepidoptera: Noctuidae) larvae used for the experiments were obtained from the Laboratory of Plague Control at the University of Caxias do Sul, Caxias do Sul, Brazil. An artificial diet based on previous work [53] was prepared consisting of 2.15 L of distilled water, 35 g of agar, 125 g of type 1 carioca bean, 100 g of wheat germ, 25 g of powdered whole milk, 62.5 g of yeast extract, 6 g of ascorbic acid, 10 mL of Vanderzant vitamin mixture, 250 mg of tetracycline, 6 mL of 40% formaldehyde, 5 g of methyl parahydroxybenzoate (Nipagin), 3 g of sorbic acid, and 50 g of soy protein. The same diet was used for larvae maintenance and bioassays. The larvae were starved for 16 h at 25 °C under a photoperiod of 16 h.day^−1^ provided by white fluorescent lamps prior to the experiment.

Bioassays with third instar insect larvae were performed using the no-choice method [54], with 6 replicates per treatment and two independent experiments. Briefly, 15 mL centrifuge tubes (Greiner^®^, 17 mm × 120 mm) were bottom-filled with 900 µL of dissolved diet and 100 µL of the following treatments: AE 4 mg.mL^−1^, AE 40 mg.mL^−1^, QB-80 2 mg.mL^−1^, QB-90 1 mg.mL^−1^, QB-90 2 mg.mL^−1^, methanol 30% (negative control), or Deltametrin 0.1 mg.mL^−1^ (K-Othrine^®^) (positive control). Tubes were left 1 h to solidify and offered to each larva. Larvae were kept for 72 h at 25 °C, 16 h light photoperiod and weighed on day zero, 1, 2, and 3.

### 4.4. Statistical Analysis

GraphPad Prism 5.0 was used for drawing graphs and statistical analyses. Results were analyzed by ANOVA followed by Tukey test, whenever appropriate, using the statistics package SPSS 20.0 for Windows. Data were expressed as mean ± standard deviation (S.D.) and significance level was *p* ≤ 0.05.

## 5. Conclusions

Examination of *Q. lancifolia* saponins for antiherbivore and antifungal activities showed that they constitute promising biological pesticides. The application of saponins is possibly a means to control different invertebrate herbivores, phytopathogens, and even human pathogenic fungi. In the future, more experiments with different herbivore models and methods of saponin application should be pursued. As some saponins may also be toxic to mammals, further studies are required to determine their safety before field application studies.

Taken together, data suggest a defense role for *Q. lancifolia* saponins, both against fungal pathogens and herbivores. In addition, potential use as an antifungal agent for human infectious mycopathogens was shown and deserves additional studies. The application of saponins, both alone and/or in combination with other compounds, should be considered as an environmentally friendly alternative for the control of plant pathogenic fungi and herbivores, reducing the need for chemical pesticides.

## Data Availability

The original contributions presented in this study are included in the article/Supplementary Materials, and further inquiries can be directed to the corresponding author.

## Supplementary Materials

The following supporting information can be downloaded at: https://www.mdpi.com/article/10.3390/plants14081252/s1, Supplementary Figure S1. Representative saponin structures present in QB fractions. A. Bidesmosidic saponin with typical glycosylations at the C-3 and C-28 positions of the aglycone nucleus (dotted circle). B. Common substituents found in ‘R’ positions of QB saponins: Fa corresponds to two units of 3,5-dihydroxy-octanoic acid; Fa-Ara has an additional arabinose residue; MeBu refers to 2-methylbutanoyl. Adapted from reference [15]. Supplementary Figure S2. Average weight of *S. frugiperda* over three days feeding on a diet containing AE and saponin fractions of *Q. lancifolia*. Diet preparation formulated with saponins fractions QB-80 2 mg.mL^−1^ or QB-90 1 or 2 mg.mL^−1^, AE 40 mg.mL^−1^, deltamethrin 0.1 mg.mL^−1^ (positive control) or methanol 30% (negative control).
