# Supplementary material for: Quillaja lancifolia Immunoadjuvant Saponins Show Toxicity to Herbivores and Pathogenic Fungi
Source: Plants (Basel). 2025 Apr 20;14(8):1252. doi: 10.3390/plants14081252 (PMC12030171; doi:10.3390/plants14081252)
Supplement: Supplementary file 1 [file plants-14-01252-s001.zip › plants-3595253-supplementary.pdf]

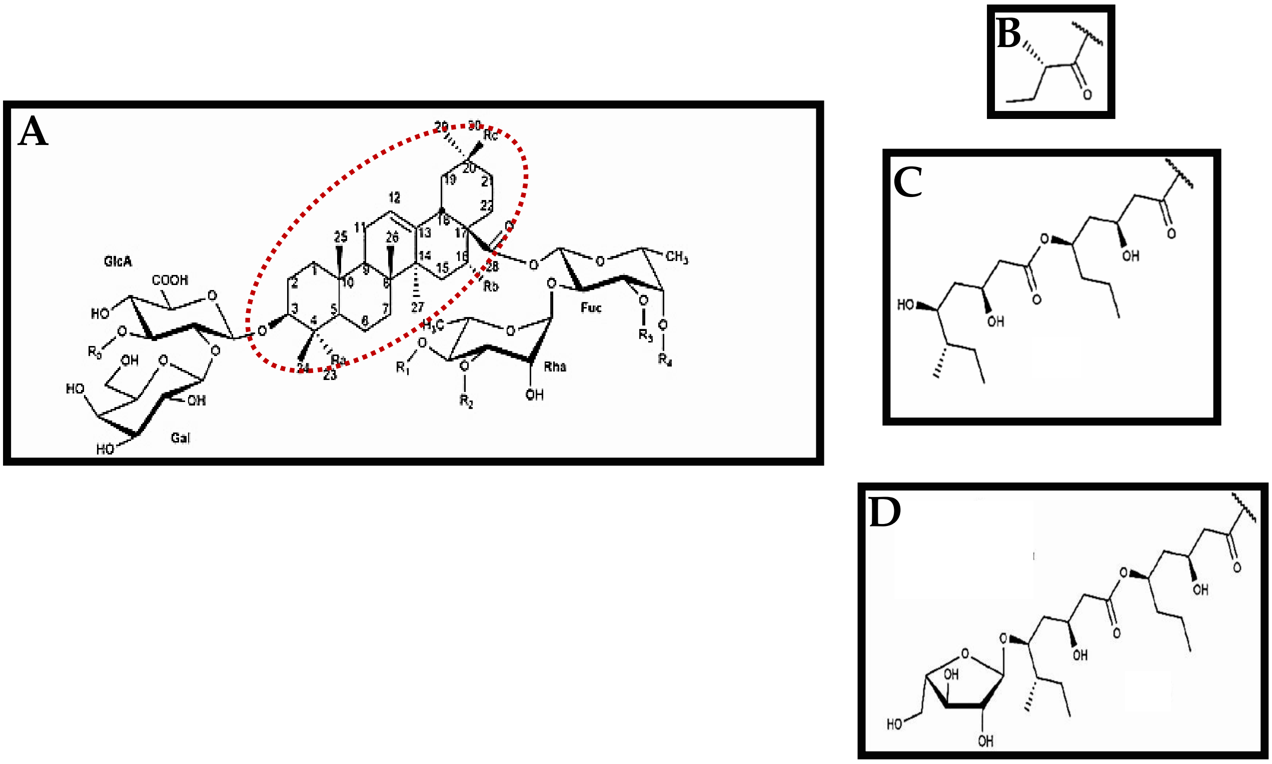

Figure S1. Representative structures present in QB saponin fractions. A. Bidesmosidic saponin with typical glycosylations at the C-3 and C-28 positions of the aglycone nucleus (dotted circle). B to D are common substituents found in 'R' positions of QB saponins: B. 2-methylbutanoyl. C. Two units of 3,5-dihydroxy-octanoic acid (Fa); D. Fa-Ara has an additional arabinose residue. Adapted from reference [15].

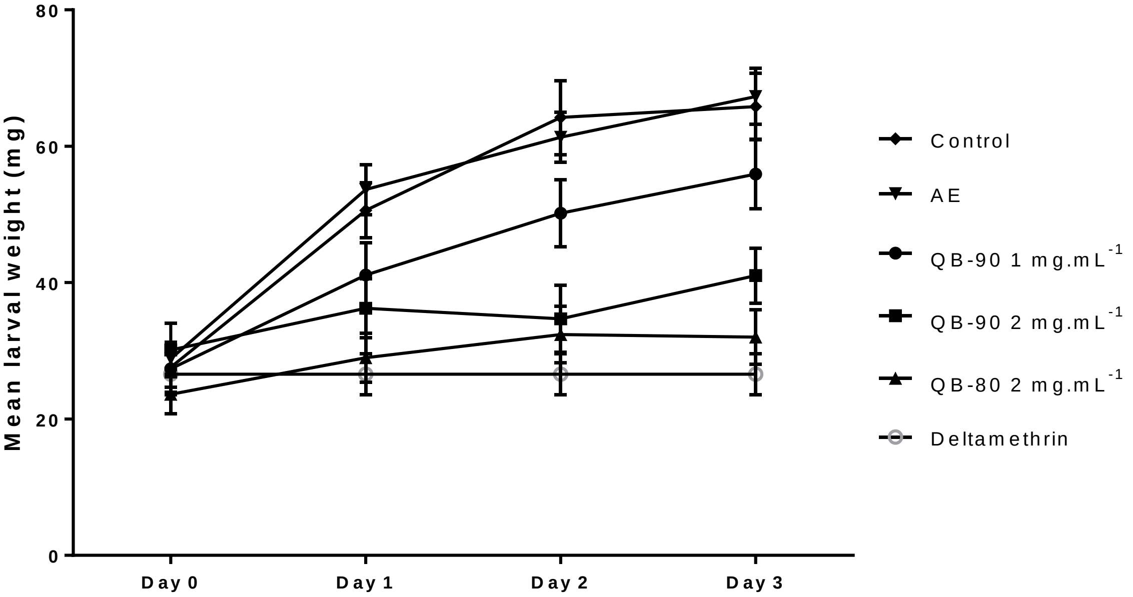

Figure S2. Average weight of *S. frugiperda* over three days feeding on a diet containing AE and saponin fractions of *Q. lancifolia*. Diet preparation formulated with saponins fractions QB-80 2 mg. mL^{-1} or QB-90 1 or 2 mg. mL^{-1}, AE 40 mg. mL^{-1}, Deltamethrin 0.1 mg. mL^{-1} (positive control) or methanol 30% (negative control).
